# Effects of Different Grazing Disturbances on the Plant Diversity and Ecological Functions of Alpine Grassland Ecosystem on the Qinghai-Tibetan Plateau

**DOI:** 10.3389/fpls.2021.765070

**Published:** 2021-12-13

**Authors:** Wenlong Li, Chenli Liu, Wenying Wang, Huakun Zhou, Yating Xue, Jing Xu, Pengfei Xue, Hepiao Yan

**Affiliations:** ^1^State Key Laboratory of Grassland Agro-Ecosystems, Key Laboratory of Grassland Livestock Industry Innovation, College of Pastoral Agriculture Science and Technology, Lanzhou University, Lanzhou, China; ^2^Department of Life Sciences, Qinghai Normal University, Xining, China; ^3^Key Laboratory of Cold Regions Restoration Ecology, Qinghai Province, Northwest Institute of Plateau Biology, Chinese Academy of Sciences, Xining, China; ^4^College of Resources and Environment, Chengdu University of Information Technology, Chengdu, China; ^5^School of Agriculture and Forestry Economic and Management, Lanzhou University of Finance and Economics, Lanzhou, China

**Keywords:** grazing management, alpine grassland, species richness, biomass, meta-analysis, Qinghai-Tibet Plateau

## Abstract

Grazing is one of the main human disturbance factors in alpine grassland on the Qinghai-Tibet Plateau (QTP), which can directly or indirectly influence the community structures and ecological functions of grassland ecosystems. However, despite extensive field grazing experiments, there is currently no consensus on how different grazing management approaches affect alpine grassland diversity, soil carbon (C), and nitrogen (N). Here, we conducted a meta-analysis of 70 peer-reviewed publications to evaluate the general response of 11 variables related to alpine grassland ecosystems plant diversity and ecological functions to grazing. Overall, the results showed that grazing significantly increased the species richness, Shannon–Wiener index, and Pielou evenness index values by 9.89% (95% CI: 2.75–17.09%), 7.28% (95% CI: 1.68–13.62%), and 3.74% (95% CI: 1.40–6.52%), respectively. Aboveground biomass (AGB) and belowground biomass (BGB) decreased, respectively, by 41.91% (95% CI: −50.91 to −32.88%) and 17.68% (95% CI: −26.94 to −8.52%). Soil organic carbon (SOC), soil total nitrogen (TN), soil C:N ratio, and soil moisture decreased by 13.06% (95% CI: −15.88 to −10.15%), 12.62% (95% CI: −13.35 to −8.61%), 3.27% (95% CI: −4.25 to −2.09%), and 20.75% (95% CI: −27.89 to −13.61%), respectively, whereas, soil bulk density and soil pH increased by 17.46% (95% CI: 11.88–24.53%) and 2.24% (95% CI: 1.01–3.64%), respectively. Specifically, moderate grazing, long-durations (>5 years), and winter grazing contributed to increases in the species richness, Shannon–Wiener index, and Pielou evenness index. However, AGB, BGB, SOC, TN, and soil C:N ratios showed a decrease with enhanced grazing intensity. The response ratio of SOC was positively associated with AGB and BGB but was negatively related to the Shannon–Wiener index and Pielou evenness index. Furthermore, the effects of grazing on plant diversity, AGB, BGB, SOC, and TN in alpine grassland varied with grazing duration, grazing season, livestock type, and grassland type. The findings suggest that grazing should synthesize other appropriate grazing patterns, such as seasonal and rotation grazing, and, furthermore, additional research on grazing management of alpine grassland on the QTP is needed in the future.

## Introduction

Grassland is an important component of terrestrial ecosystems, accounting for approximately 20% of the total global land surface (Scurlock and Hall, 1998). Grasslands play vital roles not only in supporting living and grazing conditions but also in mitigating the effects of both local and global climate change (Zhang et al., 2015; Ren et al., 2016; Yan et al., 2020). Among them, the alpine grassland on the Qinghai-Tibet Plateau (QTP) is the highest elevation grassland ecosystem in the world, at an average elevation of over 4,000 m, with over 85% of the QTP covered by alpine grasslands (Li et al., 2013; Liu X. et al., 2021). As one of the main pastoral areas in China, the QTP is abundant in grassland resources with alpine meadow, alpine steppe, and alpine desert steppe types, which occupy 47.05, 30.98, and 7.41% of the total plateau area, respectively (Tian et al., 2014; Li et al., 2018). However, because of human overuse and climate change, the alpine grassland has degraded seriously in recent decades, resulting in a loss of biodiversity and the degradation of ecosystem functions (Li et al., 2017; Liu et al., 2018). Among the factors affecting alpine grassland, grazing is regarded as one of the most important, especially overgrazing, which can lead to grassland degradation (McSherry and Ritchie, 2013). In order to mitigate further deterioration and degradation of grassland, the Chinese government has correspondingly implemented ecological restoration programs, such as the Returning Grazing Land of Grassland initiative (Zhang et al., 2015; Xiong et al., 2016). The main purpose of these programs is to reverse the negative effects of overgrazing and rebuild the ecological functions of degraded grassland areas. Since then, grazing management has become a widely effective strategy to help prevent grassland degradation and maintain sustainable grazing on the QTP (Lu et al., 2017; Liu X. et al., 2021).

To date, a number of experimental studies have been conducted to examine and clarify the impact of different grazing management strategies on plant diversity and the soil properties of alpine grassland. The outcomes of these studies are not consistent – for example, the results of Wu J. S. et al. (2014) showed that grazing reduced species diversity, whereas, Xiong et al. (2014) found that grazing for 6 years enhanced the species diversity of alpine grassland areas compared to non-grazing. Zou et al. (2016) also concluded that the plant diversity in grazing alpine grassland increased significantly compared to diversity in areas with fences. Furthermore, previous studies have generally found that grazing reduced grassland biomass (Lin et al., 2011; Zhao et al., 2016), while a few studies showed that grazing increased biomass (Niu et al., 2009). Different studies have also reported marked differences in the effects of grazing on soil properties in alpine grassland. Some studies have reported that grazing had negative effects on soil carbon (C) and nitrogen (N) levels (Sun et al., 2011; Shi et al., 2013; Li H. Q. et al., 2016), while other studies found that grazing had no significant effect on either of these parameters (Lu X. Y. et al., 2015). In addition, many previous studies have demonstrated that heavy grazing (HG) reduces plant diversity, plant biomass, and soil organic carbon (SOC) content in alpine grassland (Sun et al., 2011; Dlamini et al., 2016; Sun Y. et al., 2018) and that moderate grazing (MG) might help to balance the competing factors of species diversity protection and biomass production (Li et al., 2011). However, the results of these studies are controversial and inconclusive due to differences in grazing intensity, grazing duration, grassland types, and environmental factors between individual studies (Li et al., 2017; He et al., 2020). Consequently, to better understand the effects of differences in grazing on plant diversity and soil properties of alpine grassland on the QTP, it is urgent to conduct a systematic and comprehensive analysis based on published literature.

Meta-analysis provides a robust, quantitative, scientific, and comprehensive statistical approach to integrating information from individual studies (Hedges et al., 1999; Gurevitch et al., 2018) and several publications have synthesized the effects of grazing on grassland ecosystems at global and national scales. For example, Gao and Carmel (2020) performed a global meta-analysis and found that grazing significantly increased plant richness compared to grazing exclusion. In contrast, Herrero-Jauregui and Oesterheld (2018) reported that species richness significantly decreased as grazing intensity increased on a global scale. Zhou et al. (2017) and Byrnes et al. (2018) conducted global meta-analyses, which indicated that grazing significantly decreased belowground C and N levels in grassland ecosystems. Furthermore, Yan et al. (2013) found that, compared to the global average value, grazing had a greater negative effect on grassland total biomass in China.

However, an area-specific synthesis of the effects of grazing on alpine grassland on the QTP is still lacking and, in particular, the results of such an analysis may not be consistent outcomes of meta-analysis on a global and national scale. In terms of recent meta-analysis studies, both Yan et al. (2020) and Liu C. et al. (2021) showed that grazing significantly decreased the biomass or SOC of alpine grasslands on the QTP. However, a comprehensive analysis of the response of plant communities and soil properties of alpine grassland on the QTP to different grazing patterns remains unclear. Given these uncertainties, it is thus necessary to integrate the available data from the study area to analyze how grazing affects alpine grassland on the QTP.

In this study, we compiled data from 70 peer-reviewed studies and conducted a meta-analysis to quantitatively assess the impact of grazing on alpine grassland plant diversity indices, plant biomass, and soil properties on the QTP. Specifically, our principal objectives were (a) to evaluate the magnitude and direction (i.e., positive or negative change) of grazing disturbance on plant diversity, biomass, and soil properties in alpine grassland; (b) to examine how differences in grazing intensity, grazing duration, grazing season, livestock type, and grassland type regulate these response variables; and (c) to understand the effects of grazing disturbances on interactions between plant diversity and biomass and soil properties. These findings could provide new perspective for the formulation of grazing management strategies in alpine grasslands on the QTP.

## Materials and Methods

### Data Collection and Compilation

**FIGURE 1:**
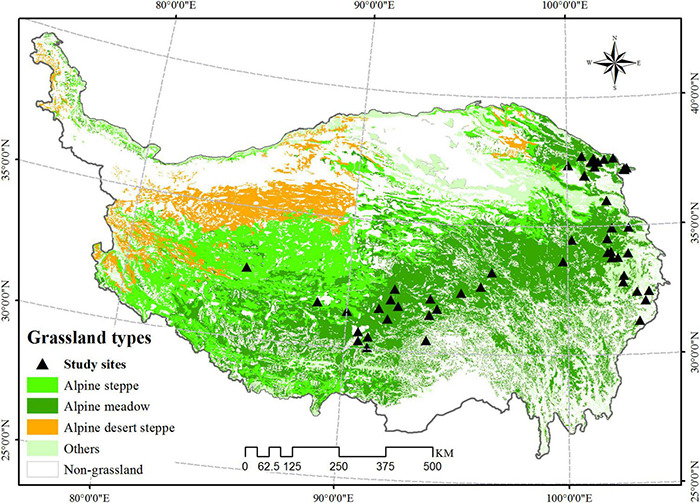
The distribution of grazing experiments selected in this meta-analysis on the QTP. The geographical location of field studies was mapped in ArcGIS 10.2 (https://www.esri.com/).

To identify we searched peer-reviewed papers published before January 2020 using the ISI Web of Science^1^ and China National Knowledge Infrastructure.^2^ The following keywords and combinations were used for retrieval: “grazing” OR “fencing” AND “alpine grassland” OR “alpine meadow” OR “alpine steppe” OR “soil carbon” OR “soil nitrogen” OR “diversity” OR “biomass” AND “Tibet” OR “Tibetan Plateau” OR “Qinghai-Tibetan Plateau.” To avoid bias in publication selection, the papers were chosen based on the following criteria: (1) all study results were from field experiments and must be carried out in the alpine grassland of the QTP; (2) there was at least one group of grazing treatment and a control group (i.e., non-grazing); (3) the experiment must contain at least one pair of target variables; (4) the grazing and control experiments both need to be carried out under similar environmental conditions, including slope, aspect, orientation, and position; (5) grazing intensity, duration, season, and livestock type need to be clearly described; and (6) the mean, standard deviation (SD) or standard error (SE), and sample size of each variable in the treatment and control group were clearly reported in the paper. Based on these criteria, a total of 70 published papers were selected for this study (Figure 1 and Supplementary Text 1).

The compiled database included three categories, comprising a total of 11 variables, as follows: (1) plant diversity – because different indices represent different aspects of species diversity, we used the common indicators of species richness, the Shannon–Wiener index, and the Pielou evenness index; (2) plant biomass, including aboveground biomass (AGB) and belowground biomass (BGB); and (3) soil properties, including SOC, soil total nitrogen (TN), soil C:N ratio, soil bulk density (BD), soil moisture (SM), and soil pH. Note that we selected BGB data in a depth range between 0 and 30 cm because more than 80% of grassland biomass and soil nutrients are concentrated in surface soil at depths of 0–30 cm (Yu et al., 2019). To detail the impact of grazing on grassland, referring to the previous studies of He et al. (2020) and Liu C. et al. (2021), based on the original papers’ data collection, the grazing levels were classified divided into light grazing (LG), MG, HG, and free grazing (FG). In addition, we also evaluated the influence of different grazing durations [short-term grazing (≤2 years), medium-term grazing (2–5 years), and long-term grazing (>5 years)] (Liu C. et al., 2021), grazing seasons (winter season, summer season, and annual grazing), and livestock types (yak, Tibetan sheep, mixed yak, and Tibetan sheep). The grassland types in each study were classified into the alpine meadow, alpine steppe, and alpine desert steppe, which are the three main grassland types on the QTP (Figure 1). All raw data were extracted from the studies’ text, tables, and graphics. If data were presented graphically, we used the GetData Graph Digitizer to extract data (ver 2.26, Russian Federation^3^). For each of the selected papers, we recorded the journal name, study site, latitude, longitude, elevation, MAT, and MAP (Supplementary Text 1). In cases where the MAT and MAP were not reported in the paper, the data were extracted from the global climate database^4^ using the study location’s corresponding latitude and longitude information (Zhou et al., 2017).

### Meta-Analysis

The data were analyzed by adopting a meta-analysis method based on Hedges et al. (1999) and Luo et al. (2006). A natural logarithm of the calculated response ratio (*RR*) was used as the effective amount to indicate the effect of grazing on the grassland-related variables. The *RR* was calculated using Eq. 1:


(1) $$RR=\ln\left(\bar{X_{t}}/\bar{X_{c}}\right)=\ln\left(\bar{X_{t}}\right)-\ln\left(\bar{X_{c}}\right)$$

where ${}_{\bar{X_{t}}}$ and ${}_{\bar{X_{c}}}$ are the mean values in the grazing treatment group and control group (non-grazing), respectively. The variance (v) of *RR* was estimated using Eq. 2:


(2) $$v=\frac{S_{t}^{2}}{n_{t}{\bar{X_{t}}}^{2}}+\frac{S_{c}^{2}}{n_{c}{\bar{X_{c}}}^{2}}$$

where *n*_*t*_ and *n*_*c*_ represent the sample sizes of the grazing treatment and the control (non-grazing) groups, respectively, and *S*_*t*_ and *S*_*c*_ are the SD of the variable of interest in the grazing treatment and control group, respectively. In order to obtain lower variability and higher accuracy, the weighted response ratio (*RR_++_*) was used to improve the statistical accuracy, with the weight factor (*w*) of the effect value (*RR*) of each study given by the inverse of its variance (*w* = *1*/*v*). The mean response ratio (*RR_++_*) was calculated from each pair of control and grazing treatments, based on individual *RR* values (Zhou et al., 2017). The equation for calculating the weighted *RR* is shown in Eq. 3:


(3) $$RR_{++}=\frac{\sum_{i=1}^{m}\sum_{j=1}^{k}w_{ij}RR_{ij}}{\sum_{i=1}^{m}\sum_{j=1}^{k}w_{ij}}$$

where *w*_*ij*_ is the weight factor for each group. The *m* and *k* values are the number of datasets and data points in each category group, respectively. The effect of grazing was considered significant if the 95% confidence interval (CI) values of *RR_++_* for a he CI is given by Eq. 4:


(4) $$95\%CI=RR_{++}\pm 1.96S\left(RR_{++}\right)$$

The overall SE (*S*) was calculated using the equation given in Eq. 5:


(5) $$S\left(RR_{++}\right)=\sqrt{\frac{1}{\sum_{i=1}^{m}\sum_{j=1}^{k}w_{ij}}}$$

We applied the random-effects model to calculate the mean effect size for each study, which used the bootstrapping method to obtain the lowest and highest values to derive the bootstrap 95% confidence interval (95% CI) based on 5,000 iterations (Janssens et al., 2010; Zhou et al., 2018). As noted above, instances where the 95% CI of *RR_++_* did not overlap with zero indicated cases where grazing had a significant impact on the selected variables. In contrast, if the 95% CI overlapped with zero, it was assumed that there was no significant difference in the variable under various grazing conditions. The percentage change of the variable was then calculated with the following equation, given in Eq. 6:


(6) $$Change\left(\%\right)=\left(e^{RR_{++}}-1\right)\times 100\%$$

To further examine the effects of categorical classes, the total heterogeneity (*Q*_*T*_) was composed of within-group heterogeneity (*Q*_*W*_) and between-group heterogeneity (*Q*_*B*_) (Ren et al., 2018). To establish whether there was a distinct difference among different treatments within the same group, if the probability value of *Q*_*B*_ was lower than 0.05, the response rates were interpreted to be significantly different among the various subgroups (Li Y. et al., 2016). The publication bias (Supplementary Text 1) was tested using Rosenthal’s fail-safe number method in the meta-analysis (Rosenberg et al., 2000; Møller and Jennions, 2001; Rosenberg, 2005). If the fail-safe number is larger than 5*n* + 10 (where n is the number of observations used in the analysis), then the result is considered to be a robust and reliable estimate of the true effect (Ren et al., 2016; Zheng et al., 2019). In addition to the above methods, we also performed Pearson’s correlation analysis to explore the relationships between the *RR* of plants and soils under grazing and the relationships between these response variables and MAT and MAP. All meta-analyses were calculated using METAWIN 2.1 software (Hedges et al., 1999; Rosenberg et al., 2000) and the plots were made using SIGMAPLOT 11.0 software (Systat Software Inc., San Jose, CA, United States).

## Results

### Effects on Grassland Plant Diversity

**FIGURE 2:**
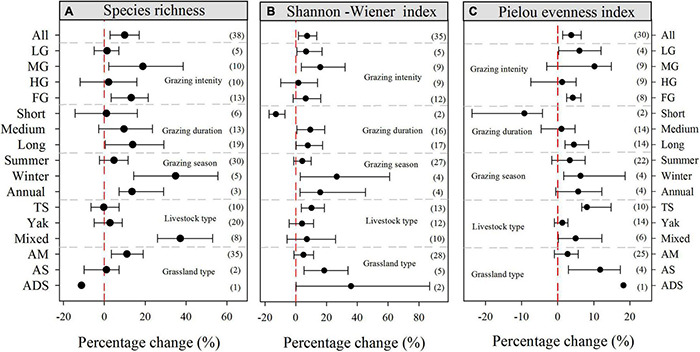
Percentage changes in **(A)** species richness, **(B)** Shannon–Wiener index, and **(C)** Pielou evenness index in response to grazing. The variables are categorized into different groups by grazing intensity, grazing duration, grazing season, livestock type, and grassland type. The error bars represent the bootstrap 95% CI. Data on the right-hand side of each panel represents the sample sizes of observations. LG, light grazing; MG, moderate grazing; HG, heavy grazing; FG, free grazing; TS, Tibetan sheep; AM, alpine meadow; AS, alpine steppe; ADS, alpine desert steppe.

Across all the observations compiled in this study, our meta-analysis showed that grazing significantly increased all the grassland diversity indices: the species richness, Shannon–Wiener index, and the Pielou evenness index increased on average by 9.89% (95% CI: 2.75–17.09%), 7.28% (95% CI: 1.68–13.62%), and 3.74% (95% CI: 1.40–6.52%), respectively (Figure 2). Among the different grazing intensities, MG had the largest impact on both species richness and the Shannon–Wiener index, increasing these values by 18.79% (95% CI: 2.08–38.65%) and 15.89% (95% CI: 3.68–32.04%), respectively; however, it did not significantly affect the Pielou evenness index (Figure 2). Furthermore, FG had positive effects on species richness (13.10%, 95% CI: 3.38–21.47%) and the Pielou evenness index (4.17%, 95% CI: 2.45–6.44%). For the experimental duration, short and medium grazing durations did not significantly increase species richness compared to non-grazing, however, long-duration grazing increased species richness by 13.83% (95% CI: 0.47–29.16%). In contrast, short-duration grazing significantly reduced both the Shannon–Wiener index and Pielou evenness index, with decreases of 12.95% (95% CI: −17.33 to −6.99%) and 9.23% (95% CI: −23.67 to −4.18%), respectively. In terms of the grazing season, winter grazing contributed to increased species richness (34.84%, 95% CI: 14.32–55.66%), Shannon–Wiener index (26.58%, 95% CI: 2.95–60.91%), and Pielou evenness index (6.31%, 95% CI: 1.67–18.67%). With respect to livestock type, grazing by Tibetan sheep significantly increased the Shannon–Wiener index and Pielou evenness index by 10.20% (95% CI: 3.63–18.55%) and 8.07% (95% CI: 6.66–14.83%), respectively (Figure 2). Grazing in an alpine meadow environment significantly increased species richness by 11.02% (95% CI: 3.50–19.08%), whereas it had no significant effect on either the Shannon–Wiener index or Pielou evenness index. In contrast, both the Shannon–Wiener index and Pielou evenness index significantly increased after grazing in an alpine steppe environment (Figure 2).

### Effects on Grassland Biomass

**FIGURE 3:**
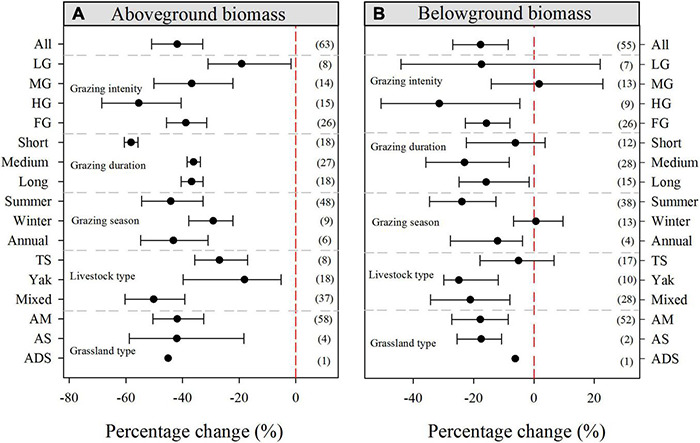
Percentage changes in **(A)** aboveground biomass and **(B)** belowground biomass in response to grazing. The variables are categorized into different groups by grazing intensity, grazing duration, grazing season, livestock type, and grassland type. The error bars represent the bootstrap 95% CI. Data on the right-hand side of each panel represents the sample sizes of observations. LG, light grazing; MG, moderate grazing; HG, heavy grazing; FG, free grazing; TS, Tibetan sheep; AM, alpine meadow; AS, alpine steppe; ADS, alpine desert steppe.

The overall responses of the selected grassland plant biomass indices to grazing are presented in Figure 3. On average, grazing significantly decreased the AGB and BGB by 41.91% (95% CI: −50.91 to −32.88%) and 17.68% (95% CI: −26.94 to −8.52%), respectively. In detail, all grazing intensities had significant negative effects on AGB, however, only heavy and FG significantly decreased BGB (Figure 3). In terms of grazing duration, short grazing duration had the greatest impact on AGB, with a decrease of 58.19% (95% CI: −60.53 to −55.71%), but did not affect BGB. Similarly, both medium and long grazing durations had significantly negative effects on the AGB and BGB parameters. Regarding different grazing seasons, winter grazing had less impact on AGB than summer and annual grazing, while it had no significant effect on BGB. Grazing by different livestock types showed different magnitudes of biomass changes, with mixed grazing showing the greatest reduction in AGB compared with yak and Tibetan sheep grazing. Furthermore, grazing significantly decreased both AGB and BGB in alpine meadow and alpine steppe environments (Figure 3). In short, grazing significantly reduced AGB, however, the values for BGB differed due to different grazing management strategies.

### Effects on Grassland Soil C, N, and Related Variables

**FIGURE 4:**
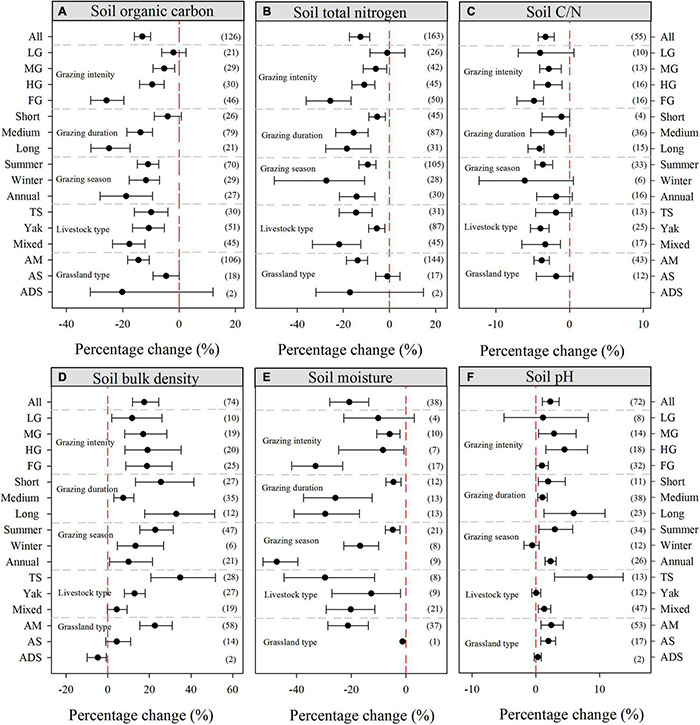
Percentage changes in soil organic carbon **(A)**, soil total nitrogen **(B)**, soil C:N ratio **(C)**, soil bulk density **(D)**, soil moisture **(E)**, and soil pH **(F)** in response to grazing. The variables are categorized into different groups by grazing intensity, grazing duration, grazing season, livestock type, and grassland type. The error bars represent the bootstrap 95% CI. Data on the right-hand side of each panel represents the sample sizes of observations. LG, light grazing; MG, moderate grazing; HG, heavy grazing; FG, free grazing; TS, Tibetan sheep; AM, alpine meadow; AS, alpine steppe; ADS, alpine desert steppe.

Averaged across all studies, grazing significantly decreased SOC (13.06%, 95% CI: −13.06 to −10.15%), TN (12.62%, 95% CI: −17.35 to −8.61%), the C:N ratio (3.27%, 95% CI: −4.25 to −2.09%), and SM (20.75%, 95% CI: −27.89 to −13.61%). However, on average, grazing increased soil BD by 17.46% (95% CI: 11.88–24.53%) and soil pH by 2.24% (95% CI: 1.01–3.64%) (Figure 4). Specifically, with increasing intensity, grazing had an increasingly negative impact on SOC, TN, C:N ratio, and SM, whereas, it had a positive effect on both soil BD and pH. Regarding grazing duration, long grazing durations had the greatest impact on SOC (−24.90%, 95% CI: −31.32 to −17.38%), TN (−18.52%, 95% CI: −27.69 to −8.12%), and C:N ratio (−4.10%, 95% CI: −5.64 to −3.48%). Moreover, all grazing durations significantly increased soil BD and soil pH, whereas they significantly decreased SM; on average, SM decreased more with increased grazing duration. In terms of grazing season, all grazing seasons significantly reduced the SOC, TN, and SM parameters, but increased soil BD. When grouped by livestock type, the greatest reductions in SOC, TN, and C:N ratio occurred with mixed livestock grazing, with decreases of 17.63% (95% CI: −23.59 to −12.08%), 21.85% (95% CI: −33.40 to −12.44%), and 3.30% (95% CI: −6.49 to −1.23%), respectively. However, Tibetan sheep grazing had the most significant impact on soil BD (+34.76%, 95% CI: 20.73–51.65%), SM (−29.61%, 95% CI: −44.57 to −11.45%), and soil pH (+8.49%, 95% CI: 2.92–13.68%). Additionally, in terms of environment, based on the limited number of observations (*n* < 20), grazing had no significant effect on the SOC, TN, or SM variables in alpine steppe and alpine desert steppe settings. However, the opposite result was identified for meadow grassland, where grazing significantly reduced SOC, TN, C:N ratio, and SM, but both soil BD and pH were significantly increased (Figure 4).

### Plant Diversity and Biomass Relationship With Soil Properties Under Grazing

As shown above, grazing affected the relationship between plant communities and soil properties in alpine grassland. In order to quantify this relationship, we used Pearson’s correlation analysis to investigate the *RR* of plant diversity, biomass, and soil environmental factors (Table 1). The results showed that the *RR* of SOC was significantly negatively correlated with both the Shannon–Wiener index and the Pielou evenness index, however, it was significantly positively correlated with AGB and BGB. In contrast, the *RR* of species richness showed no significant correlation with SOC and TN (*p* > 0.05). In addition, the relationships between the *RR* of the Shannon–Wiener index, Pielou evenness index, AGB, and BGB all had a significant negative correlation with BD (Table 1).

**TABLE 1:** Pearson’s correlation coefficients (*R*^2^) between the response ratio (*RR*) of plant and soil.

Variables	Species richness	Shannon–Wiener index	Pielou evenness index	AGB	BGB
SOC	0.01 (5)	−0.78* (5)	−0.83* (5)	0.27* (19)	0.33** (36)
TN	0.13 (16)	−0.45* (10)	−0.33 (10)	0.05 (26)	0.12* (37)
C:N ratio	–	–	–	0.04 (5)	0.87* (5)
BD	−0.45 (6)	−0.76* (6)	−0.64* (7)	−0.31** (30)	−0.20* (24)
SM	–	−0.34 (6)	−0.34 (6)	0.08 (17)	−0.37* (14)
Soil pH	0.48 (4)	0.14 (5)	0.06 (7)	−0.03 (21)	−0.01 (8)

*p < 0.05; **p < 0.01. The symbol “−” indicates no data available. Values in parentheses indicate the sample size of observations.

In addition, we also investigated the relationship between the *RR* and climate factors. Specifically, our analysis indicated that there was no significant correlation between the *RR* of plant diversity and MAT or MAP (Table 2). Similarly, no significant relationships were observed between the *RR* values of AGB, BGB, SOC, and TN and climate. The *RR* of the soil C:N ratio, SM, and soil pH were all significantly negatively correlated with MAT (*p* < 0.05), however, they were not significantly correlated with MAP. The *RR* of BD declined with increasing MAP (*p* < 0.05), however, it was not significantly correlated with MAT (Table 2).

**TABLE 2:** Pearson’s correlation coefficients (*R*^2^) between response variables, mean annual temperature (MAT), and mean annual precipitation (MAP).

Variables	*n*	MAT			MAP		
		Intercept	Slope	*R* ^2^	Intercept	Slope	*R* ^2^
Species richness	38	0.07	0.01	0.03	–0.04	2.E-04	0.02
Shannon–Wiener index	34	0.09	0.01	0.04	0.21	−2.E-04	0.05
Pielou evenness index	30	0.03	6.E-03	0.04	0.122	−2.E-04	0.035
AGB	63	–0.51	−0.02	9.E-03	–0.71	3.E-04	8.E-03
BGB	55	–0.12	−0.04	0.04	–0.10	−8.E-05	6.E-04
SOC	126	–0.16	0.02	0.03	–0.18	4.E-05	6.E-04
TN	163	–0.15	0.01	3.E-03	–0.22	1.E-04	4.E-03
C:N ratio	55	–0.05	−0.03	0.08*	0.05	−2.E-04	0.06
BD	74	0.17	0.01	0.02	0.52	−6.E-04	0.18*
SM	38	–0.25	−0.06	0.19*	0.21	−8.E−04	0.08
Soil pH	72	0.04	−0.01	0.15*	0.04	−4.E-05	0.01

*p < 0.05. n, indicates the sample size of observations.

## Discussion

### Response of Grassland Plant Diversity to Grazing

Our findings indicate that grazing significantly increases species richness, the Shannon–Wiener index, and the Pielou evenness index in alpine grassland on the QTP, consistent with the results of a meta-analysis by Lu et al. (2017) located in alpine grassland. These outcomes may be explained by grazing reducing plant height, cover, dominance, and litter, increasing light availability, enhancing the niche of grassland communities, promoting the coexistence of species, and improving plant species diversity in alpine grassland (Ren et al., 2016; Segre et al., 2016). In addition, Gao and Carmel (2020) also found that grazing significantly increased plant richness compared to grazing exclusion scenarios, based on a global meta-analysis. However, our analysis of different subgroups found notable differences – in particular, moderate and FG remarkably increased species richness, whereas, the effect was not significant in areas of light and HG. Two possible explanations have been proposed for these patterns: (1) LG has more dominant species in the grassland community to a certain extent, thus preventing the establishment of other invasive species (Ganjurjav et al., 2015); or (2) MG reduces the dominance of certain species and promotes an increase in short-statured species, thus facilitating species coexistence, while HG may eliminate some grazing-intolerant species and reduce species diversity. Additionally, Sun et al. (2021) also found that MG increased the species richness in alpine grassland, consistent with the predictions of the intermediate disturbance hypothesis, which further supports our meta-analysis results. Light, moderate, and FG significantly increased the Shannon–Wiener index and Pielou evenness index, whereas HG did not affect them. This change may be due to the decrease of dominant species in alpine grassland after grazing, which ultimately affected the plant Shannon–Wiener index and Pielou evenness index (Zhou et al., 2006; Li et al., 2013).

Notably, long-term (>5 years) grazing durations increased species diversity compared to non-grazing, however, the effects of short- and medium-duration grazing on species diversity were not significant. This may be potentially associated with the succession of alpine grassland vegetation: long-term non-grazing has led to dominant species which are more robust and, as a result, some less competitive species have gradually decreased or disappeared from plant communities because of competition, light resource, or nutrient availability (Yan and Lu, 2015; Liu et al., 2020). This result is consistent with a China scale meta-analysis performed by Xiong et al. (2016) that found that short-term grazing exclusion (<5 years) remarkably increased species richness. In addition, our results show that long-term grazing also increased the Shannon–Wiener index and Pielou evenness index. This finding is similar to that of Xiong et al. (2014), who reported that both the Shannon–Wiener index and Pielou evenness index were significantly lower in the grazing excluded plots than in the adjacent grazing plots at all sites. Moreover, our synthesis indicates that winter grazing significantly increased species richness, the Shannon–Wiener index, and Pielou evenness index, perhaps because winter grazing may reduce the accumulation of grassland litter and increase the sunshine exposure of the ground surface, thus leading to an increase in the emergence rate and number of seed species (Zou et al., 2016). In terms of livestock type, yak and Tibetan sheep are the dominant species grazing on the QTP (Cheng et al., 2016). Our meta-analysis indicated that yak grazing had no significant impact on plant diversity indices, but mixed grazing significantly increased species richness and the Pielou evenness index. It is worth noting that the effects of Tibetan sheep grazing on the Shannon–Wiener index and Pielou evenness index were more pronounced than the effects of yak and mixed grazing. This was presumably because there are differences between Tibetan sheep and yak in size and habit, including feeding and trampling behaviors (Cai et al., 2014). The increase of species richness was most pronounced in the alpine meadow environment under grazing, which could be attributed to the different components of different alpine grassland types.

### Response of Grassland Biomass to Grazing

Livestock grazing significantly decreased both AGB and BGB in the alpine grasslands of QTP (Figure 3), a finding that is supported by outcomes of many previous synthesis studies (Lu et al., 2017; Sun et al., 2021). In addition, Hao and He (2019) and Jiang et al. (2020) also found that grazing reduced AGB and BGB through China-scale meta-analysis. Among the different grazing intensities, HG yielded the most significant reductions in AGB and BGB by 55.43% (95% CI: −68.49 to −40.51%) and 31.35% (95% CI: −50.60 to −4.60%), respectively (Figure 3). This may occur because, as the destruction of soil increase as grazing pressure increases, SOC and soil nutrients decrease, and, thus, soil BD and soil pH also increase (Wiesmeier et al., 2012; Liu C. et al., 2021). Furthermore, we also found that SOC was significantly positively correlated with AGB and BGB under grazing (Table 1). However, light and MG intensities had no significant effect on BGB, which was consistent with meta-analysis on global scales (Tang et al., 2019). In addition, Yan et al. (2013), who performed a meta-analysis in China, found that light and MG did not have significant effects on grassland BGB at depths of 0–30 cm. Compared with medium and long durations of grazing, the short-duration (<2 years) grazing decreased AGB, while there was no significant change in BGB. This result is in agreement with the findings of Hao and He (2019) and may be due to the impact of short-term grazing on BGB having a certain lag effect. For different grazing seasons, grazing significantly decreased AGB and BGB, however, winter had no significant effect on BGB, which might be due to the following reasons. Firstly, summer is the growing season for alpine grassland on the QTP and grazing livestock may inhibit the normal growth of grassland by eating and trampling the plants. Secondly, the majority of grassland species stopped growing above ground in winter, however, their underground roots did not stop growing. The effect of mixed grazing on AGB was greater than that of yak or Tibetan sheep grazing, whereas, yak grazing had the greatest impact on BGB. This phenomenon might be related to the different living habits of yak and Tibetan sheep, including differences in their eating, walking, resting, and excretion habits (Cai et al., 2014). Overall, for all three grassland types, grazing reduced grassland AGB and BGB, which is consistent with findings of previous studies (Yan and Lu, 2015; Zhao et al., 2016; Chen et al., 2018).

### Response of Soil C, N, and Related Variables to Grazing

Soil C and N are materials that store energy and limit plant productivity in grassland ecosystems (Song et al., 2017). Overall, our meta-analysis indicated that grazing significantly reduced SOC, TN, and the C:N ratio in alpine grasslands, which is in accordance with the outcomes of several other studies (Lu et al., 2017; Hao and He, 2019; Zhan et al., 2020; Liu C. et al., 2021). This effect may be attributed to a decrease in grassland biomass and litter after grazing, which, in turn, leads to a decrease in the soil nutrient input (Zhou et al., 2017). With increased grazing intensity, only LG did not have a significant impact on SOC, TN, and the C:N ratio. Dong et al. (2012) and Tang et al. (2019) indicated that TN and SOC exhibited downward trends with increasing grazing intensity. These findings imply that the turnover of plant materials and excreta disruption of soil hastened the loss of soil C and N under different grazing pressures (Wu et al., 2010; Dong et al., 2012). It should be noted that the SOC, TN, and C:N ratio changed more significantly with long-durations of grazing, whereas short-duration grazing had the least effect on them. As shown in a previous study, long-term grazing reduced the input of soil organic matter in grassland (Zhang et al., 2018). Likewise, Zhou et al. (2017) also demonstrated a negative linear relationship between grazing duration and soil carbon and nitrogen from a global perspective. All grazing seasons exhibited a significant reduction in SOC and TN, however, winter and annual grazing did not change the C:N ratio (Figure 4). This was consistent with the findings of Wang et al. (2018), presumably because livestock feeding reduced the ability of the aboveground organic matter to return to the soil. Compared to yak and Tibetan sheep grazing, mixed grazing had the greatest impact on SOC, TN, and the C:N ratio in alpine grassland, which is consistent with the finding of a recent meta-analysis (Liu C. et al., 2021). This may be due to different foraging selectivity between Tibetan sheep and yak, leading to changes in the input and output of C and N by grazing. In terms of grassland types, our results indicated that grazing significantly decreased SOC, TN, and the C:N ratio in alpine meadow environments, but the results were opposite to those in alpine steppe and alpine desert steppe settings. These different results may be due to environmental factors (such as climatic conditions and soil properties) of grassland types (Sun et al., 2013). Furthermore, Yang et al. (2009) found that the plant biomass of alpine meadows on the QTP is typically higher than that of alpine grasslands and alpine desert grasslands.

Our synthesis also showed that grazing significantly increased soil BD and soil pH, but significantly decreased SM. This finding is in agreement with previous meta-analyses based on the QTP and at larger scales across China (Hao and He, 2019; Liu C. et al., 2021; Sun et al., 2021). In addition, Zheng et al. (2012) and Lu X. et al. (2015) found that grazing increased the soil BD, which led to a decrease in SM and an increase in the soil pH of alpine grasslands. We speculate that the following reasons could explain these results. Firstly, these outcomes may be attributed to frequent trampling of the soil during grazing, which leads to deterioration and consolidation of the soil structure and has a negative impact on SM (Davidson et al., 2017; Liu C. et al., 2021). Secondly, this is likely due to was likely because of the accumulation of excreta and urine from grazing livestock (Dong et al., 2012; Yang et al., 2016). Furthermore, long-duration grazing significantly increased soil pH; this effect is likely related to grazing having an additive effect on soil trampling. With respect to different grazing seasons, summer grazing had minimal effects on SM, which was likely due to precipitation on the QTP was mainly concentrated in summer, and the soil was moist. The effect of Tibetan sheep grazing on soil BD and pH was greater than that of mixed grazing, which is in agreement with a previous study (Xiao et al., 2018). Grazing significantly increased both the soil BD and pH of alpine grassland, based on our analysis of a number of studies in alpine steppe and desert steppe environments. Grazing significantly reduced soil BD in the alpine desert steppe but did not change the soil BD in the alpine steppe environment, possibly because soil properties and climatic conditions varied with grassland type.

### Regulating Mechanisms of the Grazing on Plant Community and Soil Properties

Overall, grazing is one of the most important factors affecting and regulating the vegetation and soil of the alpine grassland on the QTP (Yang et al., 2018). We found that the *RR* of SOC was negatively correlated with the Shannon–Wiener index and Pielou evenness index with increasing grazing interference. This change may be due to differences in the structure and function of vegetation communities, which cause different feedback mechanisms between them (Wardle et al., 2004). Our results also show that the *RR* of AGB and BGB were significantly positively correlated with SOC and TN, respectively (Table 1); this finding is likely because the soil C and N mainly come from grassland biomass and litter decomposition (Sun J. et al., 2018). Livestock can also increase soil hardness and pH by trampling, in addition to inhibiting the growth of grassland plants; this corresponds well with our correlation analysis, which indicates that the *RR* of soil BD and pH are negatively correlated with AGB and BGB, respectively (Table 1), consistent with the finding of Hao and He (2019). In addition, the effects of climate change will also affect the growth of alpine grassland vegetation on the QTP (Wu J. et al., 2014). In this study, we found that each plant diversity index was not significantly correlated with MAT or MAP under grazing (Table 2). This result may indicate that grazing disturbance is the main cause of changes in grassland species diversity (Collins and Barber, 1985). Suitable temperature and rainfall also help to increase soil microbial activity and accelerate soil organic matter mineralization (Ghee et al., 2013); however, our results indicate that SOC and TN are not significantly correlated with climate under grazing, potentially because grazing decreases biomass and litter, resulting in less soil organic matter and nutrients. The *RR* of the C:N ratio exhibited a negative correlation with MAT, indicating that MAT plays an important role in C:N ratio regulation (Klaminder et al., 2009). Furthermore, our results showed that the *RR* of SM and soil pH had a significant negative correlation with MAT under grazing, largely resulting from increases in soil evaporation and soil temperature by caused grazing (Wolf et al., 2010).

## Conclusion

Grazing is an important form of alpine grassland resources utilization on the QTP. Understanding the effects of various grazing type and extent on plant diversity and ecological functions in alpine grassland could provide a reference for grassland management practices. Our meta-analysis revealed that MG intensity significantly increased species richness, Shannon–Wiener index, and Pielou evenness index, indicating that MG intensity may be an effective management approach for improving the species diversity of alpine grassland on the QTP. In addition, long-duration (>5 years), winter, and mixed grazing could help to enhance grassland diversity. However, there was a greater decrease of AGB, SOC, and TN with increasing grazing intensity, among which light and MG had less impact on biomass and soil quality. Given these outcomes, this study indicates that grazing should be chosen according to local environmental conditions, in order to realize the sustainable utilization, biodiversity, and environmental protection of alpine grassland on the QTP.

## Data Availability Statement

The datasets presented in this study can be found in online repositories. The names of the repository/repositories and accession number(s) can be found in the article/Supplementary Material.

## Author Contributions

WL: conceptualization, writing – review and editing, supervision, and funding acquisition. CL: conceptualization, writing – original draft, methodology, validation, investigation, software, and formal analysis. WW and HZ: supervision and funding acquisition. YX: funding acquisition. JX: supervision. PX and HY: data collect and supervision. All authors have read and agreed to the published version of the manuscript.

## Conflict of Interest

The authors declare that the research was conducted in the absence of any commercial or financial relationships that could be construed as a potential conflict of interest. The reviewer YD declared a shared affiliation, with no collaboration, with one of the authors HZ to the handling editor at the time of the review.

## Publisher’s Note

All claims expressed in this article are solely those of the authors and do not necessarily represent those of their affiliated organizations, or those of the publisher, the editors and the reviewers. Any product that may be evaluated in this article, or claim that may be made by its manufacturer, is not guaranteed or endorsed by the publisher.

## Footnotes

1
http://apps.webofknowledge.com

2
http://www.cnki.net

3
http://www.getdata-graph-digitizer.com

4
http://www.worldclim.org
