# Supplementary material for: Effects of Different Grazing Disturbances on the Plant Diversity and Ecological Functions of Alpine Grassland Ecosystem on the Qinghai-Tibetan Plateau
Source: Front Plant Sci. 2021 Dec 13;12:765070. doi: 10.3389/fpls.2021.765070 (PMC8710682; doi:10.3389/fpls.2021.765070)
Supplement: Supplementary file 1 [file Data_Sheet_1.docx]

Supporting Information

Effects of different grazing disturbances on the plant diversity and ecological functions of alpine grassland ecosystem on the Qinghai-Tibetan Plateau

Wenlong Li^1^, Chenli Liu^1,^ *, Wenying Wang^2,^ *, Huakun Zhou^3^, Yating Xue^4^, Jing Xu^5^, Pengfei Xue^1^, Hepiao Yan^1^

^1^ State Key Laboratory of Grassland Agro-Ecosystems, Key Laboratory of Grassland Livestock Industry Innovation, College of Pastoral Agriculture Science and Technology, Lanzhou University, Lanzhou 730020, China

^2^ Department of Life Sciences, Qinghai Normal University, Xining 810008, China

^3^ Key Laboratory of Cold Regions Restoration Ecology, Qinghai Province, Northwest Institute of Plateau Biology, Chinese Academy of Sciences, Xining 810008, China

^4^ College of Resources and Environment, Chengdu University of Information Technology, Chengdu 610225

^5^ School of Agriculture and Forestry Economic and Management, Lanzhou University of Finance and Economics, Lanzhou 730020, China

Table **S1**. Fail-safe numbers of Rosenthal’s method for assessing publication bias of tested response variables in this meta-analysis.

| **Variables** | Number of studies | 5n+10 | Rosenthal's Fail-safe number |
| --- | --- | --- | --- |
| Species richness | 38 | 200 | 651.3 |
| Shannon-Wiener index | 35 | 185 | 309.6 |
| Pielou evenness index | 30 | 160 | **33.3** |
| AGB | 63 | 325 | 38164.4 |
| BGB | 55 | 285 | 1708.0 |
| SOC | 126 | 640 | 15368.2 |
| TN | 163 | 825 | 29494.4 |
| C:N ratio | 55 | 285 | 348.9 |
| BD | 74 | 380 | 7094.7 |
| SM | 38 | 200 | 3385771.1 |
| pH | 72 | 72 | 30991.4 |

Note: Bold type indicates possible publication bias.

**Table S2.** The weight response ratio and 95% CI of each subgroup in meta-analysis determining the effect of grazing on grassland plant diversity. The *Q* values of each subgroup in the meta-analysis determining the influence of grazing on plant diversity.

| **Index** | **Group** | **n** | ***RR_++_*** | **95% CI** | **Percentage (%)** | *Q_B_* | *Q_W_* | *Q_T_* |
| --- | --- | --- | --- | --- | --- | --- | --- | --- |
| Species richness | All | 38 | 0.0943 | 0.0271 to 0.1578 | 9.89 | — | — | 29.6526 |
|  | LG | 5 | 0.0126 | -0.0517 to 0.0689 | 1.27 | 3.4694 | 26.3574 | 29.8268 |
|  | MG | 10 | 0.1722 | 0.0206 to 0.3268 | 18.79 |  |  |  |
|  | HG | 10 | 0.0205 | -0.1260 to 0.1479 | 2.07 |  |  |  |
|  | FG | 13 | 0.1231 | 0.0332 to 0.1945 | 13.10 |  |  |  |
|  | Short | 6 | 0.0098 | -0.1538 to 0.1499 | 0.98 | 1.3265 | 28.2556 | 29.5821 |
|  | Medium | 13 | 0.0914 | -0.0284 to 0.2112 | 9.57 |  |  |  |
|  | Long | 19 | 0.1295 | 0.0047 to 0.2559 | 13.83 |  |  |  |
|  | Summer | 30 | 0.0456 | -0.0240 to 0.1102 | 4.67 | 11.6506** | 31.0369 | 42.6875 |
|  | Winter | 5 | 0.2989 | 0.1338 to 0.4425 | 34.84 |  |  |  |
|  | Annual | 3 | 0.1266 | 0.0704 to 0.2544 | 13.50 |  |  |  |
|  | Tibetan sheep | 10 | -0.0036 | -0.0680 to 0.0691 | -0.36 | 29.6749** | 23.6965 | 53.3714 |
|  | Yak | 20 | 0.0267 | -0.0515 to 0.0843 | 2.71 |  |  |  |
|  | Mixed | 8 | 0.3158 | 0.2316 to 0.4250 | 37.14 |  |  |  |
|  | Alpine meadow | 35 | 0.1045 | 0.0344 to 0.1746 | 11.02 | 0.4125 | 28.3840 | 28.7965 |
|  | Alpine steppe | 2 | 0.0095 | -0.1040 to 0.0704 | 0.95 |  |  |  |
|  | Alpine desert steppe | 1 | -0.1194 | - | -11.25 |  |  |  |
| Shannon-Wiener index | All | 35 | 0.0703 | 0.0167 to 0.1277 | 7.28 | — | — | 34.1291 |
|  | LG | 5 | 0.0645 | 0.0071 to 0.1571 | 6.66 | 2.8319 | 29.9093 | 32.7413 |
|  | MG | 9 | 0.1475 | 0.0361 to 0.2779 | 15.89 |  |  |  |
|  | HG | 9 | 0.0166 | -0.1034 to 0.1324 | 1.67 |  |  |  |
|  | FG | 12 | 0.0623 | -0.0167 to 0.1508 | 6.43 |  |  |  |
|  | Short | 2 | -0.1387 | -0.1903 to -0.0725 | -12.95 | 2.6763 | 28.5781 | 31.2544 |
|  | Medium | 16 | 0.0895 | 0.0067 to 0.1729 | 9.36 |  |  |  |
|  | Long | 17 | 0.0746 | 0.0027 to 0.1599 | 7.75 |  |  |  |
|  | Summer | 27 | 0.0411 | -0.0138 to 0.0957 | 4.20 | 4.7030 | 27.1135 | 31.8165 |
|  | Winter | 4 | 0.2357 | 0.0291 to 0.4757 | 26.58 |  |  |  |
|  | Annual | 4 | 0.1469 | 0.0264 to 0.3732 | 15.82 |  |  |  |
|  | Tibetan sheep | 13 | 0.0971 | 0.0357 to 0.1702 | 10.20 | 0.7726 | 35.9729 | 36.7455 |
|  | Yak | 12 | 0.0398 | -0.0429 to 0.1095 | 4.06 |  |  |  |
|  | Mixed | 10 | 0.0696 | -0.0582 to 0.2292 | 7.21 |  |  |  |
|  | Alpine meadow | 28 | 0.0478 | -0.0114 to 0.1099 | 4.90 | 3.4074 | 30.5340 | 33.9414 |
|  | Alpine steppe | 5 | 0.1689 | 0.0523 to 0.2918 | 18.40 |  |  |  |
|  | Alpine desert steppe | 2 | 0.3059 | 0.0009 to 0.6251 | 35.78 |  |  |  |
| Pielou evenness index | All | 30 | 0.0367 | 0.0139 to 0.0632 | 3.74 | — | — | 27.9676 |
|  | LG | 4 | 0.0582 | 0.0029 to 0.1136 | 5.99 | 7.9444* | 20.7090 | 28.6534 |
|  | MG | 9 | 0.0968 | -0.0306 to 0.1389 | 10.16 |  |  |  |
|  | HG | 9 | 0.0122 | -0.0771 to 0.0502 | 1.23 |  |  |  |
|  | FG | 8 | 0.0409 | 0.0242 to 0.0624 | 4.17 |  |  |  |
|  | Short | 2 | -0.0968 | -0.2701 to -0.0427 | -9.23 | 5.8055 | 22.8479 | 28.6534 |
|  | Medium | 14 | 0.0101 | -0.0467 to 0.0472 | 1.02 |  |  |  |
|  | Long | 14 | 0.0439 | 0.0204 to 0.0822 | 4.49 |  |  |  |
|  | Summer | 22 | 0.0329 | -0.0162 to 0.0733 | 3.34 | 3.6442 | 25.0092 | 28.6534 |
|  | Winter | 4 | 0.0612 | 0.0166 to 0.1712 | 6.31 |  |  |  |
|  | Annual | 4 | 0.0549 | -0.0056 to 0.1153 | 5.64 |  |  |  |
|  | Tibetan sheep | 10 | 0.0776 | 0.0645 to 0.1383 | 8.07 | 11.3900** | 17.2634 | 28.6534 |
|  | Yak | 14 | 0.0125 | -0.0091 to 0.0281 | 1.26 |  |  |  |
|  | Mixed | 6 | 0.0484 | 0.0024 to 0.1153 | 4.96 |  |  |  |
|  | Alpine meadow | 25 | 0.0267 | -0.0087 to 0.0558 | 2.71 | 0.3870 | 26.0084 | 26.3954 |
|  | Alpine steppe | 4 | 0.111 | 0.0294 to 0.16 | 11.74 |  |  |  |
|  | Alpine desert steppe | 1 | 0.1671 | - | 18.19 |  |  |  |

Note: *, *P* < 0.05; **, *P* < 0.01.

LG, light grazing; MG, moderate grazing; HG, heavy grazing; FG, free grazing. The grazing duration are classified as short grazing duration (≤2 years), Medium grazing duration (2-5 years), and long grazing duration (＞5 years)

**Table S3.** The weight response ratio and 95% CI of each subgroup in meta-analysis determining the effect of grazing on grassland biomass. The *Q* values of each subgroup in the meta-analysis determining the influence of grazing on grassland biomass.

| **Index** | **Group** | **n** | ***RR_++_*** | **95% CI** | **Percentage (%)** | *Q_B_* | *Q_W_* | *Q_T_* |
| --- | --- | --- | --- | --- | --- | --- | --- | --- |
| AGB | All | 63 | -0.5432 | -0.7116 to -0.3987 | -41.91 | — | — | 31.6976 |
|  | LG | 8 | -0.2122 | -0.6453 to -0.4138 | -19.12 | 2.7092 | 14.6023 | 17.3115 |
|  | MG | 14 | -0.4581 | -0.3718 to -0.0167 | -36.75 |  |  |  |
|  | HG | 15 | -0.808 | -0.6967 to -0.2507 | -55.43 |  |  |  |
|  | FG | 26 | -0.4913 | -0.6108 to -0.3785 | -38.82 |  |  |  |
|  | Short | 18 | -0.8720 | -0.9296 to -0.8145 | -58.19 | 181.3017** | 1314.9388 | 1496.2405 |
|  | Medium | 27 | -0.4481 | -0.4852 to -0.4111 | -36.12 |  |  |  |
|  | Long | 18 | -0.4588 | -0.5196 to -0.3979 | -36.80 |  |  |  |
|  | Summer | 48 | -0.5826 | -0.7859 to -0.3978 | -44.16 | 24.3450** | 776.2282 | 800.5732 |
|  | Winter | 9 | -0.345 | -0.4754 to -0.2506 | -29.18 |  |  |  |
|  | Annual | 6 | -0.5663 | -0.7937 to -0.3709 | -43.24 |  |  |  |
|  | Tibetan sheep | 8 | -0.3142 | -0.442 to -0.1865 | -26.96 | 315.7083** | 1446.8422 | 1762.5505 |
|  | Yak | 18 | -0.2003 | -0.507 to -0.0529 | -18.15 |  |  |  |
|  | Mixed | 37 | -0.697 | -0.9229 to -0.4971 | -50.19 |  |  |  |
|  | Alpine meadow | 58 | -0.5431 | -0.703 to -0.3936 | -41.91 | 0.0002 | 789.1089 | 789.1091 |
|  | Alpine steppe | 4 | -0.5446 | -0.8866 to -0.2026 | -41.99 |  |  |  |
|  | Alpine desert steppe | 1 | -0.5994 | - | -45.09 |  |  |  |
| BGB | All | 55 | -0.1946 | -0.3139 to -0.0891 | -17.68 | — | — | 32.4485 |
|  | LG | 7 | -0.1908 | -0.58 to 0.1983 | -17.37 | 4.3468 | 29.9588 | 34.3056 |
|  | MG | 13 | 0.0171 | -0.1526 to 0.2058 | 1.72 |  |  |  |
|  | HG | 9 | -0.3762 | -0.7053 to -0.0471 | -31.35 |  |  |  |
|  | FG | 26 | -0.1712 | -0.2569 to -0.0824 | -15.73 |  |  |  |
|  | Short | 12 | -0.0633 | -0.2537 to 0.0367 | -6.13 | 14.3072** | 268.2532 | 282.5604 |
|  | Medium | 28 | -0.2613 | -0.4428 to -0.0849 | -23.00 |  |  |  |
|  | Long | 15 | -0.1718 | -0.2851 to -0.0158 | -15.78 |  |  |  |
|  | Summer | 38 | -0.2726 | -0.4242 to -0.1338 | -23.86 | 27.9420** | 190.8439 | 218.7859 |
|  | Winter | 13 | 0.0066 | -0.0697 to 0.0918 | 0.66 |  |  |  |
|  | Annual | 4 | -0.1281 | -0.3244 to -0.0386 | -12.02 |  |  |  |
|  | Tibetan sheep | 17 | -0.0524 | -0.1972 to 0.0647 | -5.11 | 14.1444** | 207.0911 | 221.2356 |
|  | Yak | 10 | -0.2855 | -0.3554 to -0.126 | -24.84 |  |  |  |
|  | Mixed | 28 | -0.2362 | -0.4187 to -0.0832 | -21.04 |  |  |  |
|  | Alpine meadow | 52 | -0.1955 | -0.3168 to -0.0888 | -17.76 | 0.0005 | 218.7503 | 218.7503 |
|  | Alpine steppe | 2 | -0.1923 | -0.2941 to -0.1136 | -17.49 |  |  |  |
|  | Alpine desert steppe | 1 | -0.0636 |  | -6.16 |  |  |  |

Note: *, *P* < 0.05; **, *P* < 0.01.

AGB, aboveground biomass; BGB, belowground biomass; LG, light grazing; MG, moderate grazing; HG, heavy grazing; FG, free grazing. The grazing duration are classified as short grazing duration (≤2 years), Medium grazing duration (2-5 years), and long grazing duration (＞5 years)

**Table S4.** The weight response ratio and 95% CI of each subgroup in meta-analysis determining the effect of grazing on grassland soil C, N, and related variables. The Q values of each subgroup in the meta-analysis determining the influence of grazing on soil property.

| **Index** | **Group** | **n** | ***RR_++_*** | **95% CI** | **Percentage (%)** | *Q_B_* | *Q_W_* | *Q_T_* |
| --- | --- | --- | --- | --- | --- | --- | --- | --- |
| SOC | All | 126 | -0.1399 | -0.1804 to -0.1020 | -13.06 | — | — | 162.4508 |
|  | LG | 21 | -0.0204 | -0.0641 to 0.0245 | -2.02 | 64.4895** | 152.7415 | 217.2310 |
|  | MG | 29 | -0.0547 | -0.0974 to -0.0158 | -5.32 |  |  |  |
|  | HG | 30 | -0.1007 | -0.1516 to -0.0542 | -9.58 |  |  |  |
|  | FG | 46 | -0.2982 | -0.3772 to -0.2182 | -25.78 |  |  |  |
|  | Short | 26 | -0.0417 | -0.0919 to 0.0085 | -4.08 | 33.9793** | 172.9927 | 206.9720 |
|  | Medium | 79 | -0.1477 | -0.2050 to -0.0990 | -13.73 |  |  |  |
|  | Long | 21 | -0.2863 | -0.3757 to -0.1909 | -24.90 |  |  |  |
|  | Summer | 70 | -0.1173 | -0.1608 to -0.0753 | -11.07 | 5.6571 | 166.9198 | 172.5769 |
|  | Winter | 29 | -0.1254 | -0.1949 to -0.0724 | -11.79 |  |  |  |
|  | Annual | 27 | -0.2084 | -0.3294 to -0.0986 | -18.81 |  |  |  |
|  | Tibetan sheep | 30 | -0.1047 | -0.1725 to -0.0401 | -9.94 | 5.6401 | 143.355 | 148.9957 |
|  | Yak | 51 | -0.1137 | -0.1809 to -0.0539 | -10.75 |  |  |  |
|  | Mixed | 45 | -0.1939 | -0.269 to -0.1287 | -17.63 |  |  |  |
|  | Alpine meadow | 106 | -0.1558 | -0.2022 to -0.1121 | -14.43 | 5.5395 | 152.0696 | 157.6091 |
|  | Alpine steppe | 18 | -0.0472 | -0.0971 to 0.001 | -4.61 |  |  |  |
|  | Alpine desert steppe | 2 | -0.2257 | -0.3789 to 0.114 | -20.20 |  |  |  |
| TN | All | 163 | -0.1349 | -0.1905 to -0.0900 | -12.62 | — | — | 246.2211 |
|  | LG | 26 | -0.0097 | -0.0889 to 0.644 | -0.97 | 38.4248** | 271.8128 | 310.2375 |
|  | MG | 42 | -0.0618 | -0.121 to -0.0123 | -5.99 |  |  |  |
|  | HG | 45 | -0.1163 | -0.1785 to -0.0651 | -10.98 |  |  |  |
|  | FG | 50 | -0.2971 | -0.4478 to-0.1816 | -25.70 |  |  |  |
|  | Short | 45 | -0.0551 | -0.0933 to -0.0182 | -5.36 | 11.8679** | 272.9678 | 284.8357 |
|  | Medium | 87 | -0.1692 | -0.2659 to -0.0969 | -15.57 |  |  |  |
|  | Long | 31 | -0.2048 | -0.3242 to -0.0847 | -18.52 |  |  |  |
|  | Summer | 105 | -0.0995 | -0.1426 to -0.0602 | -9.47 | 13.5974** | 245.6338 | 259.2312 |
|  | Winter | 28 | -0.32 | -0.6913 to -0.1141 | -27.39 |  |  |  |
|  | Annual | 30 | -0.154 | -0.2441 to -0.0656 | -14.27 |  |  |  |
|  | Tibetan sheep | 31 | -0.1569 | -0.2457 to -0.0786 | -14.52 | 20.1492** | 257.6815 | 277.8306 |
|  | Yak | 87 | -0.0573 | -0.0946 to -0.021 | -5.57 |  |  |  |
|  | Mixed | 45 | -0.2466 | -0.4064 to -0.1328 | -21.85 |  |  |  |
|  | Alpine meadow | 144 | -0.1492 | -0.2069 to -0.0995 | -13.86 | 5.0190 | 242.4348 | 247.4538 |
|  | Alpine steppe | 17 | -0.009 | -0.0613 to 0.0442 | -0.90 |  |  |  |
|  | Alpine desert steppe | 2 | -0.188 | -0.3834 to 0.1374 | -17.14 |  |  |  |
| S:N ratio | All | 55 | -0.0332 | -0.0432 to -0.0206 | -3.27 | — | — | 45.5109 |
|  | LG | 10 | -0.0408 | -0.072 to 0.0058 | -4.00 | 1.8775 | 43.4712 | 45.3486 |
|  | MG | 13 | -0.0287 | -0.0416 to -0.0113 | -2.83 |  |  |  |
|  | HG | 16 | -0.0297 | -0.0493 to -0.01 | -2.93 |  |  |  |
|  | FG | 16 | -0.0496 | -0.0742 to -0.036 | -4.84 |  |  |  |
|  | Short | 4 | -0.0111 | -0.0380 to 0.0000 | -1.10 | 4.1083 | 41.2403 | 45.2403 |
|  | Medium | 36 | -0.0250 | -0.0544 to -0.0048 | -2.47 |  |  |  |
|  | Long | 15 | -0.0419 | -0.0580 to -0.0354 | -4.10 |  |  |  |
|  | Summer | 33 | -0.0372 | -0.048 to -0.0234 | -3.65 | 3.6896 | 41.6590 | 45.3486 |
|  | Winter | 6 | -0.0628 | -0.1311 to 0.0054 | -6.09 |  |  |  |
|  | Annual | 16 | -0.0185 | -0.0457 to 0.0037 | -1.83 |  |  |  |
|  | Tibetan sheep | 13 | -0.0187 | -0.0474 to 0.0032 | -1.85 | 2.9493 | 42.3994 | 45.3486 |
|  | Yak | 25 | -0.0405 | -0.0547 to -0.0282 | -3.97 |  |  |  |
|  | Mixed | 17 | -0.0336 | -0.0671 to -0.0124 | -3.30 |  |  |  |
|  | Alpine meadow | 43 | -0.0386 | -0.0493 to -0.0276 | -3.79 | 2.8124 | 42.5362 | 45.3486 |
|  | Alpine steppe | 12 | -0.0184 | -0.0463 to 0.0044 | -1.82 |  |  |  |
|  | Alpine desert steppe | - | - | - | - |  |  |  |
| BD | All | 74 | 0.1609 | 0.1114 to 0.2141 | 17.46 | — | — | 94.2894 |
|  | LG | 10 | 0.1104 | 0.0192 to 0.2313 | 11.67 | 0.6772 | 81.8404 | 82.5176 |
|  | MG | 19 | 0.1568 | 0.0783 to 0.2501 | 16.98 |  |  |  |
|  | HG | 20 | 0.1746 | 0.0791 to 0.3014 | 19.08 |  |  |  |
|  | FG | 25 | 0.172 | 0.0823 to 0.2685 | 18.77 |  |  |  |
|  | Short | 27 | 0.2270 | 0.1247 to 0.3463 | 25.48 | 16.9580** | 87.1074 | 104.0655 |
|  | Medium | 35 | 0.0716 | 0.0295 to 0.1177 | 7.42 |  |  |  |
|  | Long | 12 | 0.2842 | 0.1637 to 0.4143 | 32.87 |  |  |  |
|  | Summer | 47 | 0.2053 | 0.1437 to 0.2741 | 22.79 | 7.7633 | 81.7625 | 86.5258 |
|  | Winter | 6 | 0.125 | 0.0449 to 0.2379 | 13.31 |  |  |  |
|  | Annual | 21 | 0.0952 | 0.0083 to 0.1946 | 9.99 |  |  |  |
|  | Tibetan sheep | 28 | 0.2983 | 0.1884 to 0.4164 | 34.76 | 26.2786** | 82.4564 | 108.7351 |
|  | Yak | 27 | 0.1207 | 0.0771 to 0.1653 | 12.83 |  |  |  |
|  | Mixed | 19 | 0.0415 | -0.0017 to 0.0884 | 4.24 |  |  |  |
|  | Alpine meadow | 58 | 0.2038 | 0.1428 to 0.2697 | 22.61 | 11.5115** | 84.7429 | 96.2545 |
|  | Alpine steppe | 14 | 0.0418 | -0.0095 to 0.1036 | 4.27 |  |  |  |
|  | Alpine desert steppe | 2 | -0.049 | -0.1039 to -0.0072 | -4.78 |  |  |  |
| SM | All | 38 | -0.2326 | -0.3298 to -0.1449 | -20.75 | — | — | 30.5461 |
|  | LG | 4 | -0.1073 | -0.258 to 0.0298 | -10.17 | 13.2420** | 29.1551 | 42.3971 |
|  | MG | 10 | -0.0618 | -0.1129 to -0.0224 | -5.99 |  |  |  |
|  | HG | 7 | -0.0884 | -0.2828 to -0.0074 | -8.46 |  |  |  |
|  | FG | 17 | -0.4017 | -0.5418 to -0.2637 | -33.08 |  |  |  |
|  | Short | 12 | -0.0473 | -0.0760 to -0.0183 | -4.62 | 9.1331* | 35.2641 | 44.3972 |
|  | Medium | 13 | -0.2985 | -0.4692 to -0.1318 | -25.81 |  |  |  |
|  | Long | 13 | -0.3504 | -0.5259 to -0.1862 | -29.56 |  |  |  |
|  | Summer | 21 | -0.0498 | -0.0783 to -0.0221 | -4.86 | 274.5990** | 76.0640 | 350.6630 |
|  | Winter | 8 | -0.1836 | -0.258 to -0.1054 | -16.77 |  |  |  |
|  | Annual | 9 | -0.6401 | -0.7387 to -0.5028 | -47.28 |  |  |  |
|  | Tibetan sheep | 8 | -0.3511 | -0.59 to -0.1216 | -29.61 | 1.7711 | 29.8012 | 31.5723 |
|  | Yak | 9 | -0.1362 | -0.3157 to -0.0203 | -12.73 |  |  |  |
|  | Mixed | 21 | -0.226 | -0.3453 to -0.121 | -20.23 |  |  |  |
|  | Alpine meadow | 37 | -0.239 | -0.3363 to -0.1484 | -21.26 | - | - | 31.5655 |
|  | Alpine steppe | 1 | -0.0129 | - | -1.28 |  |  |  |
|  | Alpine desert steppe | - | - | - | - |  |  |  |
| Soil pH | All | 72 | 0.0222 | 0.0100 to 0.0358 | 2.24 | — | — | 69.1205 |
|  | LG | 8 | 0.0109 | -0.0511 to 0.0791 | 1.10 | 2.1594 | 29.2684 | 31.4278 |
|  | MG | 14 | 0.0279 | 0.0044 to 0.0611 | 2.83 |  |  |  |
|  | HG | 18 | 0.0437 | 0.0155 to 0.0779 | 4.47 |  |  |  |
|  | FG | 32 | 0.0095 | 0.0001 to 0.0195 | 0.95 |  |  |  |
|  | Short | 11 | 0.0189 | 0.0038 to 0.0453 | 1.91 | 21.8274** | 142.9228 | 164.7503 |
|  | Medium | 38 | 0.0102 | 0.003 to 0.0174 | 1.03 |  |  |  |
|  | Long | 23 | 0.0574 | 0.0127 to 0.1029 | 5.91 |  |  |  |
|  | Summer | 34 | 0.0292 | 0.0045 to 0.0559 | 2.96 | 1.8654 | 43.1508 | 45.0162 |
|  | Winter | 12 | -0.0056 | -0.0188 to 0.0052 | -0.56 |  |  |  |
|  | Annual | 26 | 0.0227 | 0.0143 to 0.0314 | 2.30 |  |  |  |
|  | Tibetan sheep | 13 | 0.0815 | 0.0288 to 0.1282 | 8.49 | 59.0340** | 144.7149 | 203.7489 |
|  | Yak | 12 | 0.004 | -0.0065 to 0.0078 | 0.04 |  |  |  |
|  | Mixed | 47 | 0.0128 | 0.0039 to 0.0224 | 1.29 |  |  |  |
|  | Alpine meadow | 53 | 0.0238 | 0.0082 to 0.0415 | 2.41 | 0.2376 | 45.1428 | 45.3804 |
|  | Alpine steppe | 17 | 0.0193 | 0.0073 to 0.0303 | 1.95 |  |  |  |
|  | Alpine desert steppe | 2 | 0.0029 | -0.0024 to 0.0083 | 0.29 |  |  |  |

*, *P* < 0.05; **, *P* < 0.01.

SOC, soil organic carbon; TN, soil total nitrogen; SCN, soil C: N ratio, BD, soil bulk density; SM, soil moisture. LG, light grazing; MG, moderate grazing; HG, heavy grazing; FG, free grazing. The grazing duration are classified as short grazing duration (≤2 years), Medium grazing duration (2-5 years), and long grazing duration (＞5 years)

**Text S1 Studies included in the current meta-analysis**

1. Chen J, Luo Y, Xia J, Zhou X, Niu S, Shelton S, Guo W, Liu S, Dai W, Cao J (2018) Divergent responses of ecosystem respiration components to livestock exclusion on the Qinghai Tibetan Plateau. Land Degradation & Development 29: 1726-1737.
2. Chai LR, Sun Y, Wang H, Chang SH, Hou FJ, Cheng YX (2018) Effect of yak grazing intensity on characteristics of plant communities and forage quality in gannan alpine meadow. Pratacultural Science 35: 18-26. (In Chinese)
3. Chen DD, Sun DS, Zhang SH, Tan YR, Du GZ, Shi XM (2011) Effect of grazing intensity on soil microbial characteristics of an alpine meadow on the tibetan plateau. Journal of Lanzhou University (Natural Sciences) 47: 73-81. (In Chinese)
4. Chen H (2012) response of vegetation community characteristic and soil physico-chemical properties grazing intensity on kobresia pygmaea meadow of Qinghai-Tibet Plateau. Master Dissertation of Northwest A&F University. (In Chinese)
5. Chen J, Shi W, Cao J (2015) Effects of grazing on ecosystem CO(2) exchange in a meadow grassland on the Tibetan Plateau during the growing season. Environ Manage 55: 347-359.
6. Chen J, Zhou X, Wang J, Hruska T, Shi W, Cao J, Zhang B, Xu G, Chen Y, Luo Y (2016) Grazing exclusion reduced soil respiration but increased its temperature sensitivity in a Meadow Grassland on the Tibetan Plateau. Ecol Evol 6: 675-687.
7. Dong QM, Zhao XQ, Wu GL, Shi JJ, Wang YL, Sheng L (2012) Response of soil properties to yak grazing intensity in a Kobresia parva-meadow on the Qinghai-Tibetan Plateau, China. Journal of Soil Science and Plant Nutrition 12: 535-546.
8. Fan Y, Hou X, Shi H, Shi S (2013) Effects of grazing and fencing on carbon and nitrogen reserves in plants and soils of alpine meadow in the three headwater resource regions. Russian Journal of Ecology 44: 80-88.
9. Ganjurjav H, Duan Mj, Wan Yf, Zhang WN, Gao QZ, Li, Y, Jiangcun WZ, Danjiu Lb, Guo HB (2015) Effects of grazing by large herbivores on plant diversity and productivity of semi-arid alpine steppe on the Qinghai-Tibetan Plateau. The Rangeland Journal 37: 389-397.
10. He GY (2014) The effect of fertilization gradients and grazing patterns on soil properties in the eastern of Qinghai-Tibet Plateau. Master Dissertation of Lanzhou University. (In Chinese)
11. Jia TT, Yuan XX, Zhao H, Yang YT, Luo KJ, Guo ZG (2013) Effects of Grazing Intensity on N and P Content in Dominant Plants and Soil in the Northeast Regions of Qinghai-Tibetan Plateau. Chinese Journal of Grassland 35: 80-85. (In Chinese)
12. Li F (2014) Effects of different management models on the community structure of free-living nitrogen-fixing microbes in the Alpine Meadow soil on Tibet Plateau. Master Dissertation of Lanzhou University. (In Chinese)
13. Li FX, Li XD, Zhou BR, Qi DL, Wang L, Fu H (2015) Effects of grazing intensity on biomass and soil physical and chemical characteristics in alpine meadow in the source of three rivers. Pratacultural Science 32: 11-18. (In Chinese)
14. Li HQ, Wei YX, He HD, Yang YS (2018) Effects of Grazing Density on Nitrous Oxide Effluxes in Alpine Kobresia Humilis Meadow on the Qinghai-Tibetan Plateau. Chinese Journal of Agrometeorology 39: 27-33. (In Chinese)
15. Li SQ (2014) Response of soil nutritents characteristics of alpine measow to grazing density in the northeastern edge of Qinghai-Tibetan Plateau. Master Dissertation of Lanzhou University. (In Chinese)
16. Li SQ, Wang XZ, Gao ZG, Zhuo J, Xue R, Shen YY (2013) Effects of Short-term Grazing on C and N Content in Soil and Soil Microbe in Alpine Meadow in the North-Eastern Edge of the Qinghai-Tibetan Plateau. Chinese Journal of Grassland 35: 55-66. (In Chinese)
17. Li W, Liu Y, Wang J, Shi S, Cao W (2018) Six years of grazing exclusion is the optimum duration in the alpine meadow-steppe of the north-eastern Qinghai-Tibetan Plateau. Sci Rep 8: 17269.
18. Li W, Tian FP, Ren ZW, Huang HZ, Zhang ZN (2013) Effects of grazing and fertilization on the relationship between species abundance and functional traits in an alpine meadow community on the Tibetan Plateau. Nordic Journal of Botany 31: 247-255.
19. Li W, Wu GL, Zhang GF, Du GZ (2011) The maintenance of offspring diversity in response to land use: sexual and asexual recruitment in an alpine meadow on the Tibetan Plateau. Nordic Journal of Botany 29: 81-86.
20. Lin XW, Zhang ZH, Wang SP, Hu YG, Xu GP, Luo CY, Chang XF, Duan JC, Lin QY, Xu B, Wang YF, Zhao XQ, Xie ZB (2011) Response of ecosystem respiration to warming and grazing during the growing seasons in the alpine meadow on the Tibetan plateau. Agricultural and Forest Meteorology 151: 792-802.
21. Lu X, Yan Y, Sun J, Zhang X, Chen Y, Wang X, Cheng G (2015) Short-term grazing exclusion has no impact on soil properties and nutrients of degraded alpine grassland in Tibet, China. Solid Earth 6: 1195-1205.
22. Luan J, Cui L, Xiang C, Wu J, Song H, Ma Q, Hu Z (2014) Different grazing removal exclosures effects on soil C stocks among alpine ecosystems in east Qinghai–Tibet Plateau. Ecological Engineering 64: 262-268.
23. Ma WM, Ding KY, Li ZW (2016) Comparison of soil carbon and nitrogen stocks at grazing-excluded and yak grazed alpine meadow sites in Qinghai-Tibetan Plateau, China. Ecological Engineering 87: 203-211.
24. Mao SJ, Wu QH, Li HQ, Zhang F, Li YN (2015) Effects of grazing intensity on species diversity and biomass in alpine-cold forb meadow on the Tibetan Plateau. Journal of Glaciology and Geocryology 37: 1372-1380. (In Chinese)
25. Miao FH (2015) response of plant community to stocking rate and precipitation variation in the grassland of northeastern edge of Qinghai-Tibetan Plateau. Doctoral Dissertation at Lanzhou University. (In Chinese)
26. Niu KC, Choler P, Zhao BB, Du GZ (2009) The allometry of reproductive biomass in response to land use in Tibetan alpine grasslands. Functional Ecology 23: 274-283.
27. Qiao CL, Wang JH, Ge SD, Chen DD, Zhao L, Li YN (2012) Comparison of soil properties under fencing and grazing in alpine meadow on Qinghai-Tibet Plateau. Pratacultural Science 29: 341-345. (In Chinese)
28. Ren QJ, Wu GL, Ren GH (2009) Effect of grazing intensity on characteristics of alpine meadow communities in the eastern Qinghai-Tibetan Plateau. Acta Prataculturae Sinica 18: 256-261. (In Chinese)
29. Rui YC, Wang SP, Xu ZH, Wang YF, Chen CR, Zhou XQ, Kang XM, Lu SB, Hu YG, Lin QY, Luo CY, 2011. Warming and grazing affect soil labile carbon and nitrogen pools differently in an alpine meadow of the Qinghai-Tibet Plateau in China. Journal of Soils and Sediments 11: 903-914.
30. Shang ZH, Ma YS, Long RJ, Ding LM (2008) Effect of fencing, artificial seeding and abandonment on vegetation composition and dynamics of ‘black soil land’ in the headwaters of the Yangtze and the Yellow Rivers of the Qinghai-Tibetan Plateau. Land Degradation & Development 19: 554-563.
31. Shi XM, Li XG, Li CT, Zhao Y, Shang ZH, Ma QF (2013) Grazing exclusion decreases soil organic C storage at an alpine grassland of the Qinghai-Tibetan Plateau. Ecological Engineering 57: 183-187.
32. Sun DS (2012) Studies on the effects of grazing intensity on vegetation and soil in alpine meadow on the eastern Qinghai-Tibetan Plateau. Doctoral Dissertation at Lanzhou University. (In Chinese)
33. Sun DS, Wesche K, Chen DD, Zhang SH, Wu GL, Du GZ, Comerford NB (2011) Grazing depresses soil carbon storage through changing plant biomass and composition in a Tibetan alpine meadow. Plant Soil and Environment 57: 271-278.
34. Sun G, Zhu-Barker X, Chen D, Liu L, Zhang N, Shi C, He L, Lei Y (2017) Responses of root exudation and nutrient cycling to grazing intensities and recovery practices in an alpine meadow: An implication for pasture management. Plant and Soil 416: 515-525.
35. Sun HZ (2014) Studies on the effects of Fertilization and Grazing on soil enzyme Activity in Eastern Qinghai-Tibet Plateau. Master Dissertation of Lanzhou University. (In Chinese)
36. Sun Y, He XZ, Hou F, Wang Z, Chang, S (2018) Grazing increases litter decomposition rate but decreases nitrogen release rate in an alpine meadow. Biogeosciences 15: 4233-4243.
37. Tserang D.M, Wen YL, Ai Y, Zhao HW, Chen YJ (2016) Impact of different grazing intensity on soil physical properties and plant biomass in Qinghai-Tibet Plateau alpine meadow ecosystem. Pratacultural Science 33: 1975-1980. (In Chinese)
38. Wang X, Nielsen UN, Yang X, Zhang L, Zhou X, Du G, Li G, Chen S, Xiao S (2018) Grazing induces direct and indirect shrub effects on soil nematode communities. Soil Biology and Biochemistry 121: 193-201.
39. Wang X, Yan Y, Cao Y (2011) Impact of historic grazing on steppe soils on the northern Tibetan Plateau. Plant and Soil 354: 173-183.
40. WANG XT (2010) effect of different grazing intensities on vegetation and soil physical and chemical character in alpine meadow. Master Dissertation of Lanzhou University. (In Chinese)
41. Wang Y, Hodgkinson KC, Hou F, Wang Z, Chang S (2018) An evaluation of government-recommended stocking systems for sustaining pastoral businesses and ecosystems of the Alpine Meadows of the Qinghai-Tibetan Plateau. Ecol Evol 8: 4252-4264.
42. Wei D, Ri X, Wang Y, Wang Y, Liu Y, Yao T (2012) Responses of CO2, CH4 and N2O fluxes to livestock exclosure in an alpine steppe on the Tibetan Plateau, China. Plant and Soil 359: 45-55.
43. Wei YL (2018) Response of soil microbial biomass and community structure to grazing and fencing in shrub grassland in eastern Qilian Mountains. Master Dissertation of Gansu Agricultural University. (In Chinese)
44. Wu GL, Du GZ, Liu ZH, Thirgood S (2008) Effect of fencing and grazing on a Kobresia-dominated meadow in the Qinghai-Tibetan Plateau. Plant and Soil 319: 115-126.
45. Wu GL, Li W, Zhao LP, Shi ZH (2011) Artificial Management Improves Soil Moisture, C, N and P in an Alpine Sandy Meadow of Western China. Pedosphere 21: 407-412.
46. Wu GL, Li XP, Cheng JM, Wei XH, Sun L (2009) Grazing Disturbances Mediate Species Composition of Alpine Meadow Based on Seed Size. Israel Journal of Ecology and Evolution 55: 369-379.
47. Wu GL, Liu ZH, Zhang L, Chen JM, Hu TM (2010) Long-term fencing improved soil properties and soil organic carbon storage in an alpine swamp meadow of western China. Plant and Soil 332: 331-337.
48. Wu GL, Shang ZH, Zhu YJ, Ding LM, Wang D (2015) Species-abundance-seed-size patterns within a plant community affected by grazing disturbance. Ecological Applications 25: 848-855.
49. Wu J, Shen Z, Shi P, Zhou Y, Zhang X (2014) Effects of Grazing Exclusion on Plant Functional Group Diversity of Alpine Grasslands Along a Precipitation Gradient on the Northern Tibetan Plateau. Arctic, Antarctic, and Alpine Research 46, 419-429.
50. Xie Z (2014) Response of Functional Groups of nitrifiers and denitrifiers to different grazing intensities in Qinghai-Tibet alpine meadows soil. Master Dissertation of Lanzhou University. (In Chinese)
51. Xiong D, Shi P, Sun Y, Wu J, Zhang X (2014) Effects of grazing exclusion on plant productivity and soil carbon, nitrogen storage in alpine meadows in northern Tibet, China. Chinese Geographical Science 24: 488-498.
52. Xu XY, Gao JJ, Yang L, Yang SR, Gong YF, Li MT (2018) Effects of grazing and enclosure on foliar and soil stoichiometry of grassland on the Qinghai-Tibetan Plateau. Chinese Journal of Ecology 37: 1349-1355. (In Chinese)
53. Xu YF, Yixi CM, Fu JJ, Chen H, Miao YJ, Chen J, Hu TM, Shan JG (2012) Response of plant diversity and soil nutrient to grazing intensity in kobresia pygmaea meadow of qinghai-tibet plateau. Acta Agrestia Sinica 20: 1026-1032. (In Chinese)
54. Yan Y, Lu XY (2015) Is grazing exclusion effective in restoring vegetation in degraded alpine grasslands in Tibet, China? Peerj 3: 16.
55. Yang Q, He GY, Sun HZ, Du GZ (2013) The response of soil physico-chemical property and microbial biomass to grazing on Tibetan Plateau. Journal of Gansu Agricultural University, 48: 76-81. (In Chinese)
56. Yang Z, Guo H, Zhang J, Du G (2013) Stochastic and deterministic processes together determine alpine meadow plant community composition on the Tibetan Plateau. Oecologia 171: 495-504.
57. Yang Z, Zhu Q, Zhan W, Xu Y, Zhu E, Gao Y, Li S, Zheng Q, Zhu D, He Y, Peng C, Chen H (2018) The linkage between vegetation and soil nutrients and their variation under different grazing intensities in an alpine meadow on the eastern Qinghai-Tibetan Plateau. Ecological Engineering 110: 128-136.
58. Yang ZA (2017) A study on the response of vegetation-soil system to grazing and nitrogen addition in an alpine meadow of Qinghai-Tibetan Plateau, China. Doctoral Dissertation of Northwest A&F University. (In Chinese)
59. Yang ZA, Jiang L, Xu YY, Zhan W, Zhu EX, Chen H (2017) Responses of vegetation and soil of alpine meadows on the Qinghai-Tibet Plateau to short-term grazing prohibition. Acta Ecologica Sinica 37: 7903-7911. (In Chinese)
60. Yang ZA, Xiong W, Xu YY, Jiang L, Zhu EX, Zhan W, He YX, Zhu D, Zhu QA, Peng CH, Chen H (2016) Soil properties and species composition under different grazing intensity in an alpine meadow on the eastern Tibetan Plateau, China. Environmental Monitoring and Assessment 188: 12.
61. Yi XC, Xu YF, Fu JJ, Sun YF, Ba SJ, Ni B, Hu TM, MiaoYJ (2014) Effects of grazing intensity on vegetation community and soil physicochemical properties of alpine meadow in Tibet. Journal of Northwest A & F University (Natural Science Edition) 42: 27-33. (In Chinese)
62. Zhai WT, Chen DD, Li Q, Zhao L, Liu Z, Xu SX, Dong Q, Zhao XQ (2017) Effect of grazing intensity on carbon metabolic characteristics of soil microbial communities in an alpine steppe in the regions around Qinghai Lake. Chinese Journal of Applied and Environmental Biology 23: 685-692. (In Chinese)
63. Zhang Y, Gao Q, Dong S, Liu S, Wang X, Su X, Li Y, Tang L, Wu X, Zhao H (2015) Effects of grazing and climate warming on plant diversity, productivity and living state in the alpine rangelands and cultivated grasslands of the Qinghai-Tibetan Plateau. The Rangeland Journal 37: 57-65
64. Zhao J, Li X, Li R, Tian L, Zhang T (2016) Effect of grazing exclusion on ecosystem respiration among three different alpine grasslands on the central Tibetan Plateau. Ecological Engineering 94: 599-607.
65. Zhao J, Luo T, Li R, Li X, Tian L (2016) Grazing effect on growing season ecosystem respiration and its temperature sensitivity in alpine grasslands along a large altitudinal gradient on the central Tibetan Plateau. Agricultural and Forest Meteorology 218-219: 114-121.
66. Zheng Y, Yang W, Sun X, Wang SP, Rui YC, Luo CY, Guo LD (2012) Methanotrophic community structure and activity under warming and grazing of alpine meadow on the Tibetan Plateau. Applied Microbiology and Biotechnology 93: 2193-2203.
67. Zhou HK, Tang YH, Zhao XQ, Zhou L (2006) Long-term grazing alters species composition and biomass of a shrub meadow on the Qinghai-Tibet Plateau. Pakistan Journal of Botany 38: 1055-1069.
68. Zhou TY, Gao J, Wang JN, Sun J, Xu B, Xue JY, He JD, Xie Y, Wu Y (2018) Effects of 7-year enclosure on an alpine meadow at the south-eastern margin of tibetan plateau based on community structure and soil physico-chemical properties. Acta Prataculturae Sinica 27:1-11. (In Chinese)
69. Zhu Z, Wang X, Li Y, Wang G, Guo H (2012) Predicting plant traits and functional types response to grazing in an alpine shrub meadow on the Qinghai-Tibet Plateau. Science China Earth Sciences 55: 837-851.
70. Zou JR, Luo CY, Xu XL, Zhao N, Zhao L, Zhao XQ (2016) Relationship of plant diversity with litter and soil available nitrogen in an alpine meadow under a 9-year grazing exclusion. Ecological Research 31: 841-851.
